# Anomalous Diffusion Induced by Cristae Geometry in the Inner Mitochondrial Membrane

**DOI:** 10.1371/journal.pone.0004604

**Published:** 2009-02-26

**Authors:** Valerii M. Sukhorukov, Jürgen Bereiter-Hahn

**Affiliations:** Kinematic Cell Research, Institute for Cell Biology and Neurosciences, Johann Wolfgang Goethe University, Frankfurt am Main, Germany; Griffith University, Australia

## Abstract

Diffusion of inner membrane proteins is a prerequisite for correct functionality of mitochondria. The complicated structure of tubular, vesicular or flat cristae and their small connections to the inner boundary membrane impose constraints on the mobility of proteins making their diffusion a very complicated process. Therefore we investigate the molecular transport along the main mitochondrial axis using highly accurate computational methods. Diffusion is modeled on a curvilinear surface reproducing the shape of mitochondrial inner membrane (IM). Monte Carlo simulations are carried out for topologies resembling both tubular and lamellar cristae, for a range of physiologically viable crista sizes and densities. Geometrical confinement induces up to several-fold reduction in apparent mobility. IM surface curvature per se generates transient anomalous diffusion (TAD), while finite and stable values of projected diffusion coefficients are recovered in a quasi-normal regime for short- and long-time limits. In both these cases, a simple area-scaling law is found sufficient to explain limiting diffusion coefficients for permeable cristae junctions, while asymmetric reduction of the junction permeability leads to strong but predictable variations in molecular motion rate. A geometry-based model is given as an illustration for the time-dependence of diffusivity when IM has tubular topology. Implications for experimental observations of diffusion along mitochondria using methods of optical microscopy are drawn out: a non-homogenous power law is proposed as a suitable approach to TAD. The data demonstrate that if not taken into account appropriately, geometrical effects lead to significant misinterpretation of molecular mobility measurements in cellular curvilinear membranes.

## Introduction

Diffusivity in biological membranes is an active area of research in cell biology and biophysics. Substantial progress achieved recently in microscopic techniques allowed for increased accuracy of protein mobility measurements in plasma membranes, which have led to paradigm changes in comprehension of its organization and function [1]–[4]. However, similar advancements on membranes belonging to intracellular organelles, like mitochondria and endoplasmic reticulum are still lacking. Their nontrivial topology invalidates many assumptions acceptable for data analysis in the case of the plasma membrane, often approximated as a flat infinite surface. Here, computer simulations may simplify the choice of the proper experimental strategies and correct interpretation of results.

Biologists recognize high mobility of the mitochondria regardless of their structural complexity. Their dynamics is considered to be essential for functional integrity of the organelles and thus for the cell viability. Fusion and fission are important events in the life of a mitochondrion and at least one function of these processes is sharing all the components within a chondriome [5]. This principle has been assumed to delay malfunction during aging [6], [7]. Spreading of proteins within a chondriome has been found to occur in the range of a few hours [8]. Diffusion of the components is a fundamental process accompanying whole-organelle dynamics on a much shorter temporal scale.

**Figure 1:**
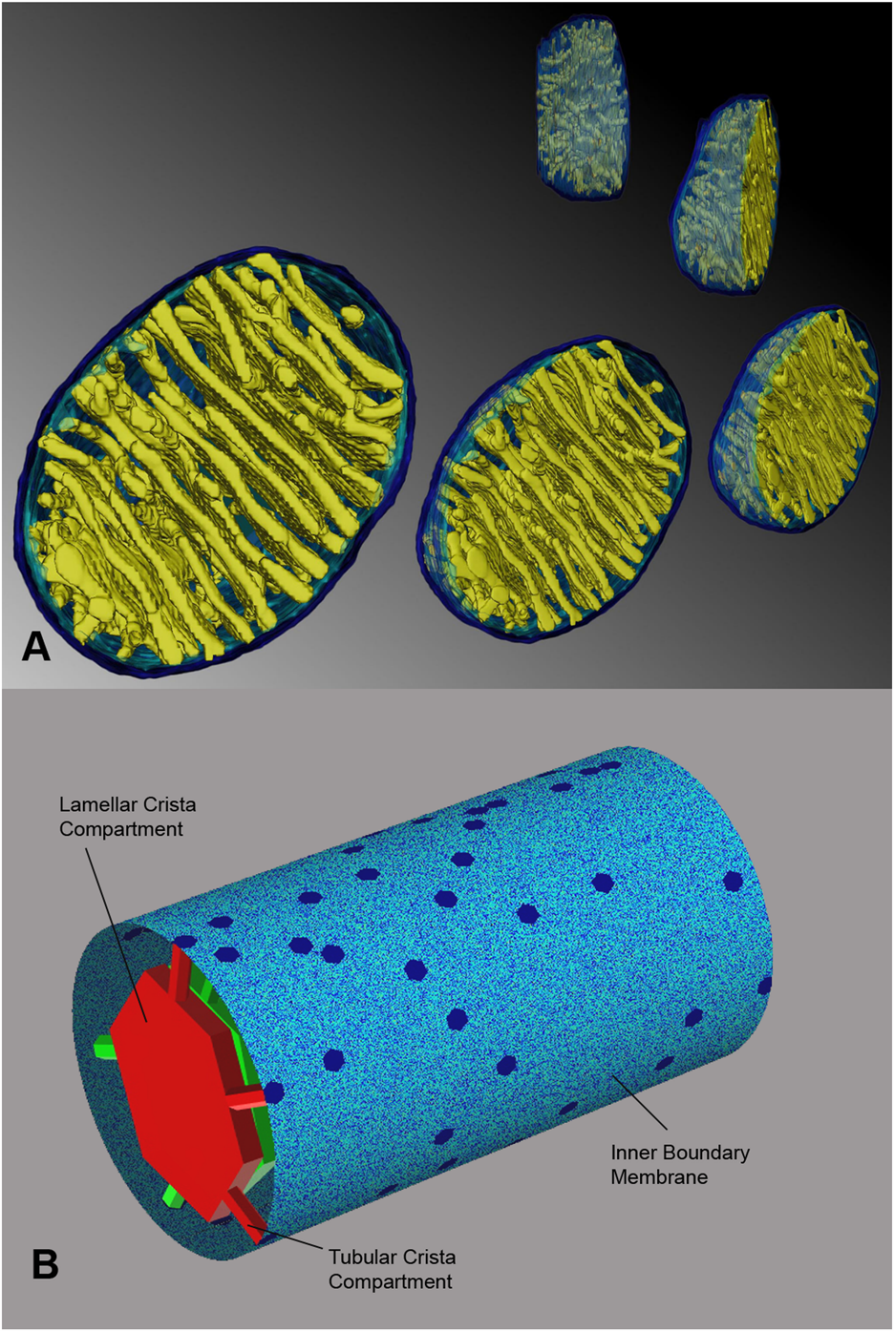
The structure of mitochondrial membraneous compartments. (*A*) Membrane surface rendering of mitochondrial tomogram. Cristae (*yellow*), inner boundary membrane (*light blue*) and outer membrane (*dark blue*) are shown from different perspectives. Image courtesy of Terrence G. Frey (San Diego St. Univ.). (*B*) Computational model of the inner mitochondrial membrane applied in this study. *Blue*: inner boundary membrane; *red* and *green*: two examplary cristae. In both (*A*) and (*B*) lamellar compartments (central cristae parts) are connected to the inner boundary membrane through a number of tubular compartments of variable lengths but of uniform diameter.

It is known since the 1950s, that mitochondria are cylindrically shaped organelles, their silhouette being formed by closely apposing outer and inner membranes, with numerous invaginations, termed cristae within the latter [9], [10]. The cristae forming mitochondrial inner membrane (IM) is contiguous with the peripheral inner membrane, referred to as inner boundary membrane (IBM). Cristae have distinctly different shapes: some organisms and cell types are known to have exclusively tubular cristae formed as curved cylinders of uniform diameter [11], others may exhibit flat or even prismatic cristae. However, in the majority of cells in multicellular organisms, cristae appear as flat membranous infoldings protruding into the mitochondrial body. We will refer to such cristae as lamellar ones. In the 1990s, electron microscopic tomography allowed for more accurate determination of their shape and connectivity to the IBM [12]–[14]. When reconstructed in 3 dimensions (Fig. 1*A* and figures in [13]), several structural features common to different tissues and species appeared (for a good review see [15]). It was found, that rather than forming folds lamellar cristae should be represented as flattened cisterns of uniform width attached to the IBM with several narrow tubular connections up to hundreds of nanometers long. Attachment places, the junctions, have a diameter (≈28 nm) similar to crista width (≈27 nm). Often, especially in smaller cristae, the flattened part is missing, so that the whole crista consists of the tubular compartment alone. When the flattened segment is present, one crista usually is anchored via several tubular connections. In tissues with large cristae masses, these are densely packed into stacks of parallel cristae, large lamellae mostly oriented perpendicular to the longer mitochondrial axis. In the extreme case of brown adipose tissue tubular compartments are totally missing, but the lamellar parts are still connected to the IBM via short junctions 28 nm in diameter.

In cellular membranes, a nontrivial dependence of diffusivity *D* on time *t* was predicted theoretically and confirmed experimentally [2], [16]–[19]. Such a diffusion pattern is called anomalous, because it exceeds the transport model represented by Fick's second law. Often, diffusivity is approximated as a power function. Consider, for example,
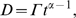
(1)where *α* is called the anomalous diffusion exponent and *Γ* is a constant. When *α* = 1, *D* = *Γ* is equal to the classical diffusion coefficient, while subdiffusive processes encountered in biological systems are characterized by *α*<1. Eq. 1 is valid for pure anomalous diffusion, in the limit *t*→*∞* only, nevertheless it was successfully applied to molecular motion in biological membranes [18].

Cellular diffusion is often discussed in connection to effects related to the presence of molecular interactions, like binding or influence of obstacles [20]–[23]. Yet, the complicated geometry of the IM may create an additional impact on diffusivity. Intuitively, it is clear that membrane infoldings reduce the diffusion coefficient projected on the mitochondrial axis. In the present study we apply a random walk model to protein diffusion within the mitochondrial IM with the goal to answer the following questions: How big is the extent of this reduction for a typical mitochondrion? Does it depend on the observation time scale? How can the diffusivity within the IM be quantified? Which parameters of crista geometry have the biggest impact on particle displacement?

Considering the lateral resolution limit >150 nm of a standard confocal laser microscope, the projection of diffusion on the long mitochondrial axis is the parameter of interest: radial diffusion would be outside this resolution limit. In the case of mitochondria, this parameter is both apparent and real. Experimental methods for the determination of macromolecular diffusivity, as fluorescence recovery after photobleaching (FRAP) or single particle tracking (SPT) determine diffusion on the basis of apparent mobility within the focal depth, about 600 nm thick, which is in the range of the thickness of a single mitochondrion. Accurate analysis and interpretation of such measurements should make possible the correct reconstruction of distortions introduced during data acquisition. Because the long mitochondrial axis is the natural route of molecular motion e.g. for material exchange after fusion, this projected mobility has a direct physiological meaning.

## Methods

### The random walk

Diffusion in the IM is simulated as a classical random walk of non-interacting point tracers on a triangular lattice. Tracers are placed at random on lattice nodes and move every time step in random direction, according to the node's connectivity.

**Figure 2:**
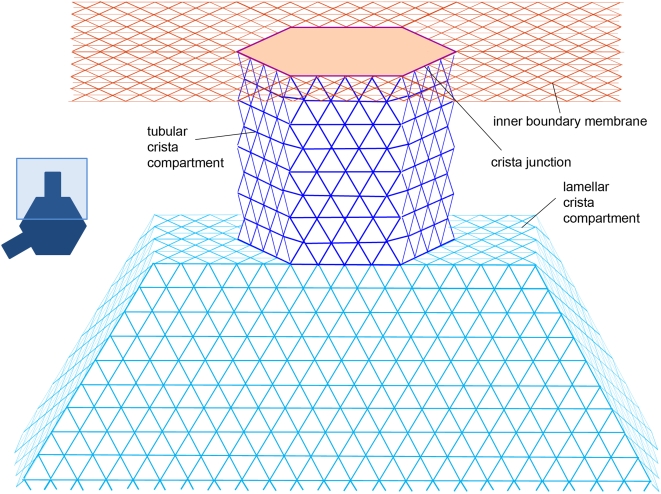
Lattice architecture of lamellar cristae. Tracers are positioned at nodes of the triangular lattice. *Red*: Inner boundary membrane, *violet*: Crista junction, *blue*: Tubular crista subcompartment, *turquoise*: Crista main body.

For mitochondria, the movement of particles along the mitochondrial long axis is physiologically relevant. Thus, for IM-based movements the effect of cristae is the determining parameter and their influence on particle diffusivity will be investigated. For every time step t*, we calculate longitudinal positions of all tracers and mean squared displacement (MSD) projection on that axis 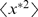 relative to the particle's initial position. The effective (projected) diffusivity is defined as
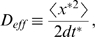
(2)where *d* is a space dimensionality. For a flat membrane in 2 dimensions *d* = 2, but if the projected diffusion along the mitochondrial axis only is considered, movements in a 1D space adequately represent protein behavior in the IM and thus *d* = 1. Since we are exclusively concerned about the impact of cristae, the use of normalized effective diffusivity *K*≡*D_eff_/D_0_*, is more convenient. Here, *D_0_* is a diffusivity projected on the same axis in a cylindrical surface containing no cristae (a base plane). For the triangular lattice shown in Fig. 2, *D_0_* = (2/3*D_lat_*, 1/3*D_lat_*) along and around the cylinder respectively, where *D_lat_* is a diffusion coefficient on the lattice. The value of *K* is discussed in the following sections. For brevity, we will refer to it as apparent diffusivity, remembering that in fact it is a ratio of two diffusion coefficients and is a dimensionless quantity.

Data are reported versus dimensionless time steps t* rather than versus experimental time *t* measured in seconds. A value of each time step *τ* can be used for conversion *t* = τt* in a way similar to conversion of dimensionless space steps r* into experimental units of length *r* = λr*, where *λ* is a lattice constant. Then,
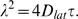
(3)


The advantage of this approach is its universality: for example, upon fixation of *λ*, *D_lat_* can still be scaled with *τ*, so that the results do not depend on the actual value of *D_lat_*.

One should remember that a lattice-based model of liquid system diffusion is an approximation. Its most important limitation is an artificial discretization of space and time. If spatial structures are modeled on the lattice, they introduce an additional scale and should be treated with care when their size is comparable to the lattice constant. For that reason, we apply variable lattice resolution depending on the time scale of interest (short-term diffusion requires higher resolution, according to Eq. 3). Subsequently, all the data are rescaled back to the largest *τ* (corresponding to a lattice bond length *λ_max_*≈0.79 nm) for the purpose of analysis and visualization.

For every set of parameters, typically, 40 to 50 different membrane configurations were generated. For MSD calculations, 10^5^ tracers uniformly distributed among nodes were used per configuration. For the calculation of the escape time distribution, 10^6^ tracers per random walk were initially placed on cristae junction nodes only. The motion of tracers that have fallen into cristae was simulated till their first return to the junction, and the time spent on crista nodes was counted. The pseudo-random number generator used is a combination of two separate congruential generators as employed in the RANDOM_NUMBER subroutine of Intel Corp. (Santa Clara, CA) Fortran compiler v.10. Data were fitted with the Levenberg-Marquardt method as implemented in MINPACK set [24].

### The inner membrane model

In order to be able to model the great diversity of IM shapes and sizes, a modular construction was assembled consisting of interconnected small flat lattice segments (Figs. 1*B*, 2). The IBM was represented by a lattice with periodic boundary conditions (PBC) in both dimensions. Application of PBC in the radial dimension is a simple consequence of the cylindrical shape of mitochondria. Since mitochondria are known to form dynamic filamentous networks *in vivo*, with their length exceeding the mitochondrial radius by orders of magnitude, the PBC in the longitudinal dimension is also a necessity. In most cases, the lattice extent in this dimension is chosen so that one replica contains 100 to 600 cristae, sufficient for eliminating finite-size effects.

**Figure 3:**
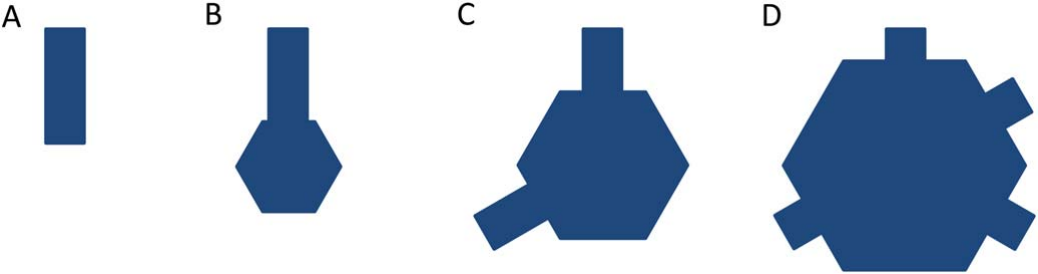
Examplary cristae configurations. (*A*) tubular; (*B*)–(*D*) lamellar.

The IBM lattice contains hexagonal areas inaccessible to tracers (shown as darker patches in Fig. 1*B*), which are surrounded by a single hexagonal layer of nodes representing IBM-crista junctions with radius *a* (*violet* in Fig. 2). When a tracer reaches the junction, it has the chance of dropping into the tubular crista compartment represented as a hexagonal cylinder of variable length *L*, connected to the IBM lattice at the junction nodes. If tubular cristae are modeled, the cylinder wall is connected to a flat lattice piece at the end opposite to junction. It represents tube's butt-end and its geometry is equal to the forbidden lattice patch of the IBM. Instead of the butt-end, the tube can be connected to a hexagonal lamellar compartment. It is represented as shallow hexagonal cylinder whose axis coincides with the longer mitochondrial axis and whose width is equal to the tube diameter as is depicted in Fig. 2. The lamella radius *R_lam_* is variable, the lamella itself is assumed to remain centered on the mitochondrial axis. This means that the radius of the mitochondrion is *R_m_* = *L*+*R_lam_*, which allows us to easily connect up to 6 tubular compartments to form a lamellar crista, exploiting the hexagonal symmetry. In the case of less than 6 tubes per crista, the sides of lamella connected to the IBM are chosen at random for each crista. Additionally, in order to avoid periodicity, each crista as a whole is rotated randomly around the long mitochondrial axis. By varying geometric dimensions of the IM lattice components, plenty of different cristae configurations can be created (Fig. 3). For simulations of variable junction permeability, lattice bonds connecting cristae tubular compartments to the IBM-tube junction where switched off with prescribed probability.

The main emphasis in the discussion below is put on membranes exhibiting tubular cristae. Despite their relative simplicity, they allow pinpointing the majority of dynamic patterns present also in mitochondria with lamellar cristae, whilst the analysis of apparent diffusivity is much easier. Since in the model the tubular cristae are assumed to be straight cylinders, their geometry is described by two parameters: radius *a* and length *L*. The radius of the tubular compartment in real cristae has been determined to be close to 13–14 nm irrespective the species or tissue types under investigation [15]. Therefore, the value 16*λ_max_*≈13 nm is used as the unit of *a*. In order to reveal the functional dependence of diffusion on the crista radius, we consider values of *a* equal to ⅛, ¼, ½ and 1 units. The tube lengths *L* are measured as a part of mitochondrial radius *R_m_* taken to be 200 nm. We examine *L* = 0.2, 0.4, 0.8, 1.6 and 2.1 mitochondrial radii. The last value is useful for comparison of tubular and lamellar topology, because tubular cristae of such length have the same surface area as lamellar ones with *R_lam_* = *R_m_*/2, for *a* = 1 and junction number 3. For lamellar cristae, we use lateral dimensions *R_lam_* = (2/8, 3/8, …, 7/8) *R_m_*. Although lamellae of real cristae have usually sizes exceeding half of the mitochondrial radius, the wider range of *R_lam_* used in this study would allow us to better compare lamellar cristae to tubular ones, which have smaller surface areas. Number of cristae junctions for the lamellar cristae is between 1 and 6. Naturally, tubular cristae have only one junction: for that reason we increase their density appropriately when comparing them to lamellar geometry. Cristae density *σ* is highly variable among different cell types, but has no influence on membrane topology, which is defined exclusively by cristae shape. As we will see later, *σ* is merely a scaling factor for the reduction in the diffusion coefficient, which prompted us to intentionally exaggerate the density over its typical physiological values, taking a close to maximal possible packing value of 42 cristae per micrometer of mitochondrial length as a standard *σ* for the lamellar topology. (With the hexagonal cylinders on a triangular lattice, Fig. 2, the maximal packing of non-contacting cylinders of radius *a* = 16 lattice steps corresponds to the distance between their centers 2(*a*+1)cos(*π*/6)*λ_max_* = 0.023 µm, *i.e. σ_max_*≈43 cr./µm.) For tubular topology, 42≤*σ*≤252 is considered, as several tubular cristae can be expected to share the same mitochondrial segment. This way, a lamellar topology with 3 junctions per crista can be compared to a tubular topology having *σ* = 126 cr/µm. Such approach is suitable, because it makes the presentation of the topology effects clearer while the diffusivities calculated using the above values of *σ* can be easily rescaled to the desired densities found in particular tissues of interest.

## Results

### Monte Carlo diffusivities

**Figure 4:**
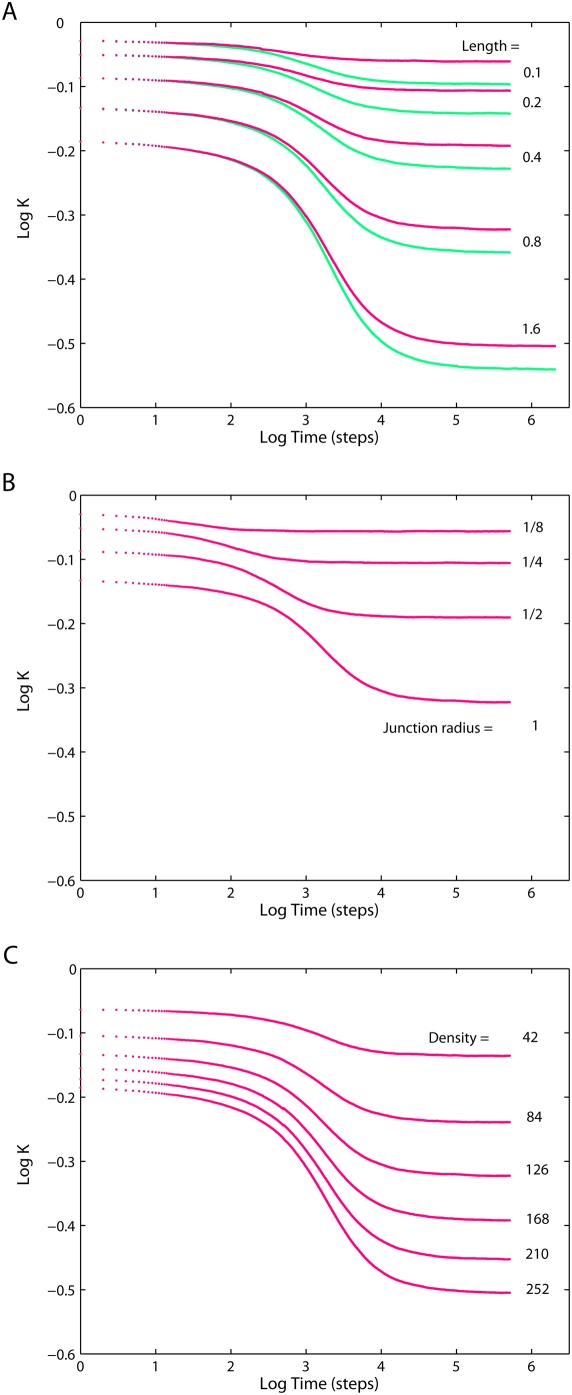
Diffusion in the inner membrane having tubular cristae. Relative diffusivities projected on the long mitochondrial axis for different tubular cristae configurations. *Red*: fully permeable junctions (p = (1,1)), *green*: fully impermeable junctions (p = (0,0)). (*A*) For indicated cristae lengths *L* (in units of mitochondrial radius *R_m_* = 200 nm). Cristae junction radius *a* = 14 nm and density *σ* = 126 cristae per µm of mitochondrial length. (*B*) For indicated cristae junction radii, (in units of *a* = 14 nm), *L* = 0.8*R_m_*, *σ* = 126, p = (1,1). (*C*) For indicated cristae densities, *a* = 14 nm, *L* = 0.8*R_m_*, p = (1,1).

**Figure 5:**
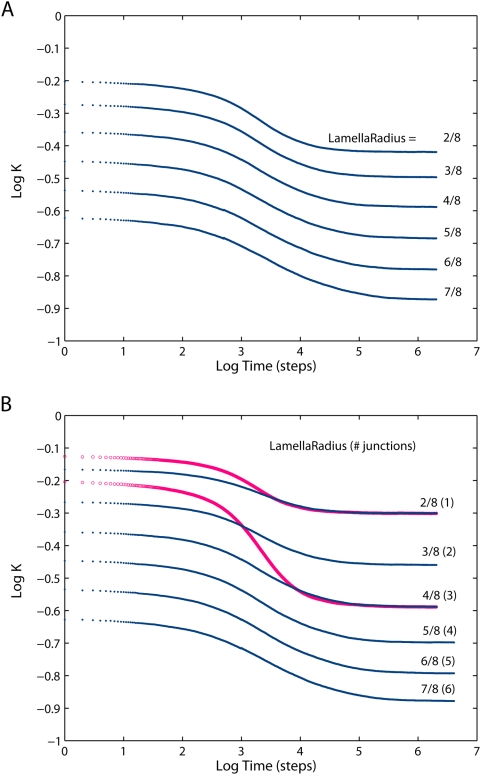
Diffusion in the inner membrane having lamellar cristae. (*A*) Relative diffusivities projected on the long mitochondrial axis for mitochondria having lamellar cristae with varying geometry as indicated by the radius of the lamellae expressed as a fraction of mitochondrial radius (*R_m_* = 200 nm) and 3 junctions per crista. Cristae junction radius *a* = 14 nm, density *σ* = 42 cristae per µm of mitochondrial length, fully permeable junctions. (*B*) *Blue dots*: same as (*A*), but for the number of junctions increasing with lamella radii from 1 to 6. This condition reflects the proposition [13] that lamellar cristae may have formed via fusion of a number of tubular ones For comparison, the projected diffusivities of two tubular geometries are shown as *red circles*. For tubular cristae, length *L* = (2.10*R_m_*, 2.18*R_m_*) and density *σ* = 126 were chosen to give the same cristae surface area and number of junctions as in the case of corresponding lamellar cristae.

Normalized projected diffusivities in the mitochondrial inner membrane are shown in Fig. 4 and Fig. 5: Apart from the overall reduction of *K*, the presence of cristae transiently induces anomalous diffusion (the nonzero slope of the curve, cf. Eq. 1). Diffusion tends to be normal in both short-time and long-time limits (referred to as *K_short_* and *K_long_* respectively). Extent of anomalous diffusivity demonstrates the dependence on both the dimensions of cristae and their density: enlargement of the junction radius (Fig. 4*B*) causes a particularly strong shift of the anomalous diffusion period towards larger *t*. Fig. 4*A* should be compared to Fig. 5*A*, since in both cases we change the main geometric parameter: *L* for the tubular cristae and *R_lam_* for the lamellar ones. Increase in those factors is equivalent to higher relative cristae surface area. If the total membrane area of tubular and lamellar topology is made equal by the appropriate setting of the length *L* of tubular cristae, identical *K_long_* are achieved, but *K_short_* is much higher for the tubular cristae (Fig. 5*B*, *circles*).

On the basis of observations by electron microscopic tomography that the number of cristae junctions is roughly proportional to lamellar size, it was proposed that lamellar cristae are the result of the merger of several tubular ones [13], [15]. A series of diffusivities shown on the Fig. 5*B* reflects this suggestion and illustrates the diffusivity changes upon smooth transition from quasi-tubular geometry (tubular cristae with a small lamellar bulge in its free end) in the upper curve to extremely lamellar ones similar to those observed in neuronal and brown adipose tissues [13]. In the latter configuration, the diffusion is reduced most strongly.

How can the above dependencies be generalized? In the discussion of the following two sections, a uniform area density of tracers was assumed, as in the MC calculations presented above. For that reason we can simplify notation by omitting weighting factors associated with relative tracer concentrations.

### Area scaling law for limiting values

It was shown analytically [25] that in a 1D space the apparent diffusivities are described exactly by an area scaling law. An analytical proof for a 2D space is not known, while the numerical data available in the literature are inconclusive, reporting conformance with both the area scaling and a differing effective medium approximation [26], [27]. This has prompted us to investigate the area scaling as a possible approach for the limiting IM diffusivities discussed above.

In the model of tubular cristae, IM area *A* per crista (measured in lattice nodes, Fig. 2) can be represented as a sum of areas corresponding to the IBM *A_ibm_* (with positions of cristae on the membrane being random, *A_ibm_* can be taken as the total IBM area divided per number of cristae), crista junction (i.e. nodes connecting tubular compartment to the IBM) *A_cj_*, tube wall *A_t_* = 6*aL* and butt-end *A_c_* : *A* = *A_ibm_*+*A_cj_*+*A_t_*+*A_c_* . The related base plane area is *A_b_* = *A_ibm_*+*A_cj_*+*A_c_*. In the long-time limit, the ratio
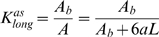
(4)is an area scaling law for mitochondria with tubular cristae, presenting dependence on cristae radius *a*, length *L* and density of cristae *σ* (through *A_b_*).

Contrary to the long-time scale where tubular walls act as a delaying potential field and individual cristae are not distinguishable, on the short-time scale (*K_short_*), on average half of tracers in the tube wall contributes to the observable mobility (tracers positioned on surfaces parallel to the long mitochondrial axis are seen as moving without restriction then, while tracers positioned on the perpendicular surfaces are seen as immobile). Hence, an area-scaling law for the short-term limiting diffusivity is:
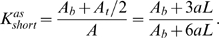
(5)


From Eq. 4 and Eq. 5 one gets for tubular cristae 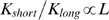. Applying similar arguments, one can calculate the values of 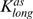 and 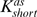 for lamellar topology as well.

**Figure 6:**
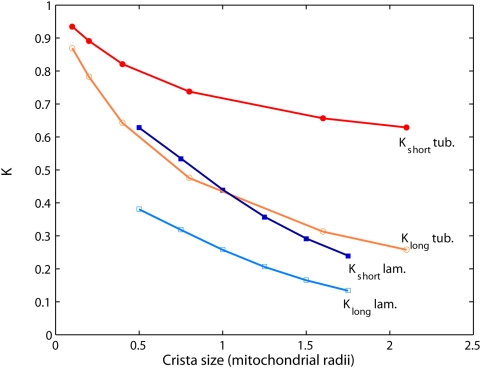
Limiting values of projected diffusivities: comparison of the MC results to the area scaling theory. Long-term (*open markers*) and short-term (*filled markers*) limiting values for tubular (*circles*) and lamellar (*squares*) cristae topologies obtained from fits to the Monte Carlo simulations (Figs. 4, 5) for different cristae sizes (*i.e.* cristae length in the case of tubular topology, lamellae diameter in the case of lamellar one), fully permeable junctions and *a* = 14 nm. Other paramemters are as in Fig. 4*A* and Fig. 5*A*. Statistical errors (40 configurations) are in the range from ±0.001 to ±0.004. The same variables computed according to the area scaling model (Eqs. 4, 5) are shown as *lines*.

For both types of cristae, theoretical values 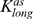 and 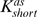 are given in Fig. 6 (*lines*) demonstrating excellent agreement with limiting diffusivities *K_long_* and *K_short_* of the simulated random walks (*circles* and *squares*) presented in the previous section. Since the expressions for long-term projected diffusivities do not depend on particular surface topology, when the lamellar and tubular membranes have equal areas, *K_long_* overlap (Fig. 5*B*).

Qualitatively, the area scaling has a simple explanation: the larger the membrane area normal to the direction of projection is, the more particles find themselves forced to move in the normal direction for some time, reducing projected MSD, and hence, 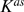. Because the area of surface projection to a mitochondrial axis normal is always more than 0 for the curved membrane, 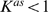 at any point on time axis.

### Dependence on time

**Figure 7:**
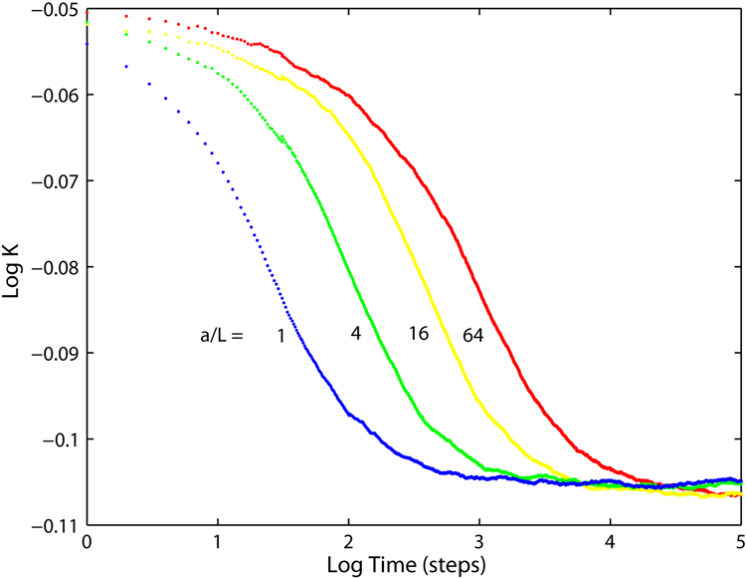
Relative projected diffusivities for tubular cristae of the same membrane area. Ratios of junction radius *a* to crista length *L* are as indicated. Cristae density *σ* = 126 cristae per µm of mitochondrial length, fully permeable junctions.

One can use the above observations for construction of a simple model for the time-dependent diffusivity *K*(*t*) in mitochondria with tubular cristae. The problem is comprehended best if we consider cristae with the same side surface area *A_t_* but different radii *a* (tube length *L* is decreased with increasing *a* to keep *A_t_* constant). Then, (Fig. 7) *K_long_*, *K_short_* and the subdiffusion exponent remain constant, while anomalous diffusion is shifted towards longer times with increasing *a*. The whole membrane can be considered as composed of cylindrical tube walls and domains parallel to the base plane. Because for both limiting values *K*(0) = *K_short_* and *K*(∞) = *K_long_* the impact of domains is determined solely by their relative areas (Eqs. 4, 5), we can assume that this is true for any *t* and approximate *K*(*t*) from a superposition of domain areas weighted with their relative projected diffusivities:
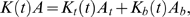
(6)where 
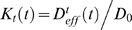
 is the diffusivity on the cristae side surface and *K_b_* is the diffusivity on the base plane, *K_b_*(*t*) = 1.

The diffusion equation for a standalone infinitely long cylinder of radius *a* can be solved exactly. Projected diffusivity in the direction perpendicular to its axis is:
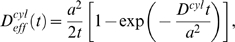
(7)where 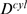 is a local diffusion coefficient on the cylinder surface [28]. For *t*→0 the apparent diffusion is normal (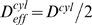), because no structure is sampled on a small time scale. In the long-time limit (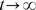) 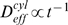, due to a finite size of the cylinder surface projection.

Upon substitution 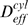 from Eq. 7 for 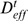, in Eq. 6:
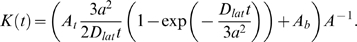
(8)


Diffusion coefficients are 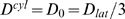 in the lattice representation. The initial period resembling normal diffusion is determined by the characteristic time 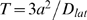. Eq. 8 has Eqs. 4 and 5 as limits for 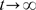 and 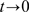 respectively, but additionally, for *t*≫*T*, one obtains the non-homogenous power-law:
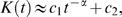
(9)where *α* = 1, 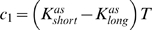 and 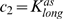 are constants.

**Figure 8:**
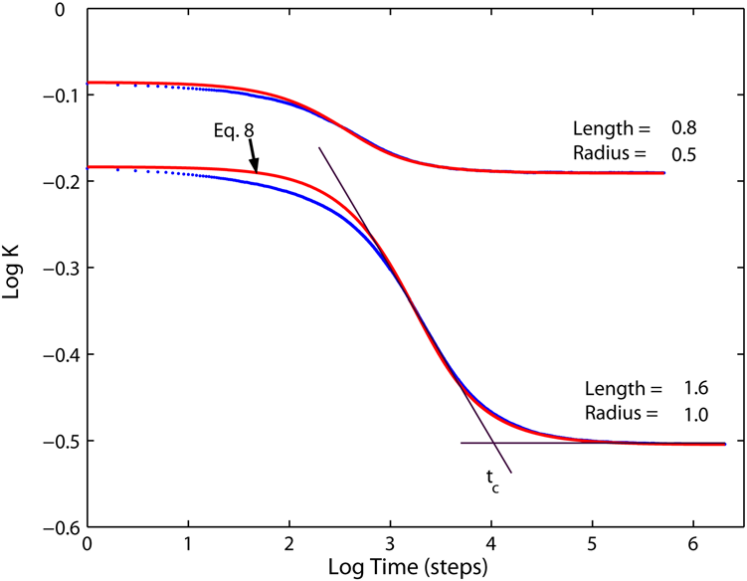
Time dependence of the projected diffusivities. Comparison of MC results for tubular cristae geometry (*dots*) to the theoretical model, Eq. 8, (*red lines*) for two examplary membrane configurations. Cristae density *σ* = 126 cristae per micrometer, fully permeable junctions. Definition of the transition time for alternative models of transient anomalous diffusion is illustrated with *black lines*.

Eq. 8 can be compared to the Monte Carlo results obtained in membranes with cristae having finite lengths as is shown in Fig. 8 for exemplary configurations. Generally, for *L*/*a*≫1 Eq. 8 offers a good approximation to the simulation data everywhere except the transition region *t*∼*T* (the discrepancy is probably due to the hexagonal cross-section of cristae in the MC lattice model). However, because Eqs. 7 and 8 assume infinitely long tubes, for cristae with *L*/*a*∼1 the exponent *α* in Eq. 9 differs from 1 decreasing the applicability of Eq. 8 (the deviation is in the range of several percent). In real membranes with tilted or curved cristae, the effective radius should be taken bigger than the tube's radius, resulting in a longer range of the short-time diffusivity regime. Even though Eq. 8 was introduced as a model for the tubular geometry only, the MC results (Fig. 5) indicate that the functional shape of Eq. 9 (with different values of parameters) can be valid for both tubular and lamellar cristae geometry.

**Figure 9:**
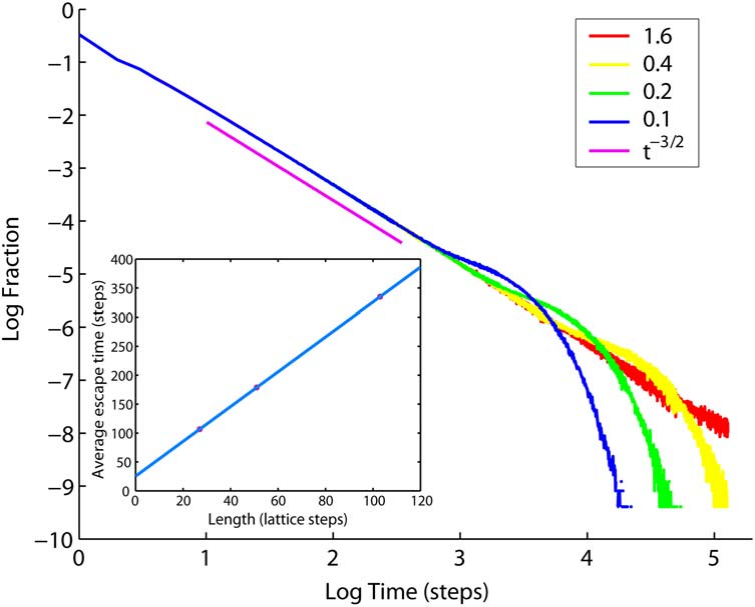
Probability distribution of escape times from tubular cristae. Cristae have radius *a* = 14 nm and lengths as indicated in the legend (in units of mitochondrial radius *R_m_* = 200 nm). Power law *t* ^−3/2^ (*magenta*) is shown for comparison. Insert: Average time spent inside a crista versus cristae length (*circles*), linear fit (*line*).

The dynamics revealed by the MC simulations and Eq. 9 is different from the pure anomalous diffusion, for which 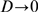 when 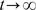. The diffusion coefficient in the inner membrane is a function of time asymptotically decreasing to a constant >0 and can be characterized as a transient anomalous diffusion (TAD). The origin of such behavior can be understood by considering the distribution of delays induced by cristae for particles diffusing along the IM, which is the same as distribution of times needed for an escape from a single crista shown in Fig. 9. Pure anomalous diffusion would be possible if this distribution had an infinite mean value 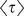, i.e. decayed slower than ∼*t* ^−2^ for 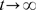 [29]. Indeed, the distribution of escape times from cristae decays as ∼*t* ^−3/2^ initially, before the particle that has dropped into a crista starts feeling the butt-end, yet for large *t* limited size of the crista membrane introduces an exponential cutoff leading to finite 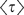 proportional to *L* (insert in Fig. 9). Such a pattern of delays generates anomalous diffusion transiently for finite *t*, which is bigger than zero: 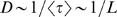 (cf. Eqs. 2, 4), but no full immobilization occurs for 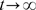. This dynamics is similar to that of a well-known 1D comb structure, for which TAD has been demonstrated also by analytical methods [29]–[33].

### Dependence on junction permeability

Although the inner mitochondrial membrane is known for decades to be an irregular but contiguous surface [9], the impact of tubular junctions connecting cristae and the IBM on protein mobility between the two domains and along the organelle remains poorly understood. It was speculated, that narrow junctions may serve as a barrier, both in restricting diffusion and in separating membrane compartments with different protein compositions [12], [13], [34]. Recent studies of protein distributions among cristae and IBM showed significant differences in concentrations of different protein species over the IM subcompartments [35], [36].

With MC simulations the role of cristae junctions in molecular diffusivity can be investigated by changing their permeability **p**: When permeability (taken the same in both directions to and from crista, i.e. symmetrically) is varied over the whole range between **p**≡(*p_in_*, *p_out_*) = (0, 0) (not permeable) and **p** = (1, 1) (fully permeable), identical apparent mobility is obtained in the short-term limit and clearly noticeable although quite restricted effect is recorded for *K_long_* (Fig. 4*A*). Evidently, decrease of junction permeability leads to a limited breakup of the area scaling law for *K_long_*, Eq. 4, but not for *K_short_*, Eq. 5.

The utilized procedure makes possible monitoring of tracer densities in each of the IM subcompartments during the same runs which were used for the acquisition of data on diffusivity dynamics. Because the tracers are represented by points designed not to interact with each other, one can expect that the non-planar membrane geometry alone would not cause differential distribution of such particles between cristae and the IBM, which is confirmed by the simulations. On the other hand, when the junction permeabilities were set to different values in the directions to and from cristae (representing one-way bottlenecks), the system has adjusted itself from the initially uniform distribution to an equilibrium at which the particles had to spend more time in the compartment with higher inwards junction permeability, raising the concentration there. This result confirms the proposition [13], [15], [34] that the cristae junctions may function as selective transporters or channels in redistributing protein species between the IM subcompartments and that such selectivity alone is sufficient for creating the protein gradients. Notably, in order to fulfill this task, the protein complexes constituting junctions must have embodied relatively sophisticated dynamic machineries able to selectively vary their directional permeability depending on the interaction with specific protein cargos. The computational verification of this possibility would require the knowledge of details of such interactions and exceeds the scope of the current study.

However, the above result raises the question concerning variation (at equilibrium) in apparent diffusion due to the asymmetry of junction permeability. As an illustration for the behavior of *K*, let us consider two extreme values of **p**, pumping the particles into either the IBM or cristae compartments. In the former case, **p**→(0, 1) hardly allowing the particles to enter cristae, which gives *K*≈*K_b_* = 1 for both short and long time regimes, similar to the situation discussed in [21], because *A_ibm_*≈*A_b_* and junction density in mitochondria is much below the percolation threshold. In the latter scenario (**p**→(1, 0), which almost certainly locks the particles inside cristae), the long-term diffusion is infinitesimally small, because the duration of anomalous diffusion regime 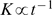 (cf. Eq. 7) is very large. Hence, the asymmetric permeability of cristae junctions is able to strongly modulate *K_long_*, inducing substantial deviations of diffusivity relative to the symmetric **p**. In this scenario, if values of **p** are different for specific protein components of the IM, this should induce not only their sorting into the membrane compartments, but also a considerable spread in observable diffusion rates.

## Discussion

Pure anomalous subdiffusion is a mathematical concept describing dynamics in disordered systems [37]. Presence of any disorder results in a series of hindrances or long-range correlations that a particle is subjected to during its motion. Because biological organisms contain numerous disorder-inducing systems, anomalous diffusion is well suited for the description of molecular motion within them. However, as their understanding advances it becomes clear, that for every disordering mechanism the subdiffusion should be expected in a narrow range of temporal and/or spatial scales determined by the particular factor involved, rather than being universal. As a consequence, the anomalous dynamics is transient, i.e. restricted to certain temporal and spatial ranges, while outside of this range classical Brownian dynamics is valid. An example is the transient anomalous diffusion (TAD) in the plasma membrane [2], [3], resulting from the compartmentation by rows of cytoskeleton-attached transmembrane proteins, hindering the motion of free particles. The semi-permeable compartment walls introduce disorder in a restricted temporal range of scales determined by their spatial structure. In a different situation, transient binding to standalone proteins with a limited choice of affinities is able to induce this kind of slowdown [38], [39]. In the current study, we show that a similar pattern of dynamics is also valid for molecular motion in the mitochondrial inner membrane (IM), where cristae delay particles on their way along the mitochondria. Here too, the extent of anomalous phase is limited and is governed by the spatial dimensions of the hindering structures, i.e. cristae. However, the case discussed here is distinct, because the anomality results from the membrane spatial curvature alone, without a necessity for any interaction with third-party objects. Such a general cause of anomalous dynamics prompts us to predict the TAD also for other types of curved cellular membranes, such as endoplasmic reticulum (ER) or the plasma membrane of cells possessing numerous microvilli.

### Implications for observability of diffusion in the non-planar membranes

Dependence of diffusivity on time has important implications for the correct design and interpretation of experimental procedures. Anomalous diffusion means that the outcome of diffusion measurements essentially depends on the time scale probed. Consider for example a particle moving with a “bare” diffusion coefficient (i.e. diffusion coefficient in a plane surface) *D_lat_* = 1 µm^2^/s in a mitochondrial inner membrane having *R_m_* = 200 nm and containing on average 21 lamellar cristae/µm, *R_lam_* = 100 nm and 3 junctions of radius 14 nm per crista. Then, one MC step is (Eq. 3) *τ*≈(0.79·10^−3^)^2^/(4·10^−6^)≈0.156 µs and diffusivity measurements investigating time scales less than ∼15 ms (or higher for tilted cristae) should demonstrate sensitivity to the probed time extent, giving *K* between *K_long_*≈0.41 and *K_short_*≈0.56 for the same sample.

TAD is characterized in terms of transition time *t_c_* quantifying the crossover between anomalous and normal diffusion regimes [21], [22], [38]. Transition time is defined as a point, at which two straight lines fitted to regions corresponding to anomalous and to normal regimes intersect on a double logarithmic plot, as is shown schematically in Fig. 8. Anomalous diffusion is postulated to be valid for *t*≪*t_c_*, crossing over to normal diffusion when *t*≫*t_c_*. Such approximation naturally corresponds to the restricted scale range of subdiffusive behavior. Yet, it may lead to inaccurate data analysis if the time window of the experimental method used for the diffusion measurement is not narrow enough. This is the case with such extensively applied methods as FRAP and fluorescence correlation spectroscopy (FCS). In a typical FCS measurement session, dynamics corresponding to several orders of magnitude in time is recorded in the same curve [Wei03]. If the anomalous diffusion is short-ranged, so that both normal and anomalous regimes are recorded, the conventional data analysis assuming either normal or anomalous diffusion will fail. Our results for diffusion in the IM show that transient anomalous diffusion should be parameterized with a non-homogenous power-law (Eq. 9), rather than with anomalous or normal regimes (which correspond to the two terms of Eq. 9) treated separately. In the latter case, the estimated ‘normal diffusion’ coefficient or subdiffusion exponent could be strongly biased.

The above conclusion was made possible because our simulation scheme was designed without any connection to particular experimental techniques used for studies of molecular mobility. Several earlier studies simulate the impact of geometry on the outcome of diffusion measurements. Sbalzarini *et al.* [40], [41] modeled molecular mobility in the ER as examined with FRAP, and compared the outcome with non-confined diffusion. They found a several-fold reduction of the diffusion coefficient in comparison to a flat space and communicated theoretical arguments in favor of anomalous diffusivity in such geometries, but did not present direct evidence based on the FRAP curves. Weiss and coworkers [19] studied diffusion in ER membranes, both experimentally and *in silico*. They found that the diffusivity in real membranes is anomalous and carried out MC simulations of FCS, specifically investigating the influence of membrane shape on the experimental outcome. However, the results of data analysis on their simulated FSC curves were ambiguous. Fractal analysis of the curves suggested purely normal diffusivity; simultaneously, in correlation analysis the anomalous diffusion model gave a better fit than the normal one, indicating dependence of the subdiffusion exponent on surface shape. Our MC results and the discussion in the previous paragraph provide an explanation for the ambiguity: Because the spatial curvature induces transient anomalous diffusion, neither pure anomalous nor normal diffusion models are suitable for the analysis of data obtained in a wide range of temporal scales. Thus, we propose a non-homogenous power law as a suitable candidate for correct description of TAD by experimental data analysis. Contrary to FCS or FRAP, single particle tracking (SPT) does not require a specific diffusion model for data analysis because it explicitly measures the particle displacement as a function of time. As a consequence, with utilization of a sufficiently wide set of sampling time frames SPT was able to successfully resolve TAD on several occasions [1], [2], [42].

Mitochondrial membrane compartments contain the highest protein concentration among biological membranes [43]. The slowdown in diffusion due to membrane curvature considered here is very likely to be enhanced by other known sources of decrease in *D*, among which crowding, corralling and aggregate formation are plausible candidates [22], [23], [39], [44], [45]. Moreover, mounting data points to a possible interplay between these factors: for instance, oligomerization of F_1_F_0_ ATP synthase particles is believed to be capable of inducing a strong bending of the IM [46]. Because conventional methods for the measurement of diffusion coefficient determine the cumulative effect due to all of the factors involved, the experimental approaches available at this time do not allow discriminatory estimation of the impact, each of these aspects has on diffusion in the IM [47]–[50], which greatly hinders understanding of their biological roles. The current study offers a simple and reliable way for decoupling the effect of membrane geometry from the accompanying sources of delay profiling the empirically determined diffusion coefficient. In order to achieve this, the measurements of molecular mobility should be supplemented with the assessment of membrane spatial structure with electron tomography. Then, application of the scaling law provides accurate correction factors related to the membrane curvature. As pointed out in the previous section, for cristae-rich mitochondria their magnitude is substantial.

### Quantitative verification of structural properties of the IM

There is growing experimental evidence that the complicated shape of the IM results from structuring by protein supercomplexes embedded in the membrane rather than being random or spontaneous [15], [34]. This organization may be expected to include a regulative mechanism adjusting molecular diffusion along the organelle through a geometrical restructuring of the membrane. Computational modeling provides the quantitative assessment for effectiveness of such a mechanism due to the possibility of explicit modulation in the values of particular membrane parameters. In the case of uniformly distributed particles, the moderate dependence of *K_long_* on cristae dimensions and density (Eq. 4, Fig. 6) implies that a radical IM reconstruction, like the one occurring during osmotic stress or apoptosis, would be required in order to achieve a noticeable change in the speed of diffusion. Which factors could be considered as plausible candidates for the role of more efficient diffusion modulators? Intuitively, the permeability of cristae junctions can be expected to serve as the crucial parameter: the junctions are the only places where direct exchange of membrane content between cristae and IBM could take place. However, our result indicates that rather than the absolute value of permeability itself, the difference in conductivity to and from the cristae (called here asymmetry) is the important aspect, because of its potential to generate protein gradients [34]. The asymmetric junction permeability was utilized here as the most straightforward way to simulate the observed differential distribution of protein complexes among IM subcompartments [35], [36]. The actual role of cristae junctions in this redistribution is the matter of experimental verification, as is the possible involvement of alternative factors like protein oligomerization or inhomogeneous presence of lipid rafts. Independently of its direct cause, the concentration gradient has a strong impact on the apparent diffusion of proteins along mitochondria. Unlike the case of uniformly distributed particles, preferential localization of protein molecules to cristae radically augments the impact of membrane topology on diffusion speed of such particles: because of a small absolute diffusivity of cristae-locked molecules, even gentle membrane restructuring would be sufficient to induce profound changes in *K_long_*. Hence, the localization to cristae can be viewed as a powerful amplifying factor with respect to geometrical changes.

The above dependence can also be exploited in experimental studies of the IM organization and function. With the long-term diffusivity being highly responsive to preferential location of particles in either cristae or IBM, one of the straightforward possibilities is an interpretation of apparent (measured) diffusion coefficient as an indicator of the partitioning: for example, a probe concentrated in the IBM would tend to diffuse quicker than that having equal density in both cristae and the IBM. Alternatively, an independent estimation of the probe distribution among membrane subcompartments should be helpful in the interpretation and analysis of diffusion data. In any case, structural properties of the IM and other curved biological membranes cannot be ignored in consideration of diffusivity on their surfaces.

### Conclusions and perspectives

Inner membrane motility is an important factor in the “rescue hypothesis” [7], [51] assuming protein exchange within the whole chondriome of a cell to stabilize functionality. Spreading of mitochondrial proteins within the chondriome has been shown experimentally for vertebrate cells in culture beyond doubt [8]. This raises the question for the parameters determining these exchange processes. Using a Monte Carlo model of mitochondrial inner membrane we have calculated geometrically-induced reduction in apparent diffusivities in the direction of the mitochondrial axis for a set of physiologically feasible configurations. Confinement resulting from the presence of cristae in the inner membrane of mitochondria imposes profound changes on the molecular mobility along the organelle. In cristae-rich cell lines, a several-fold reduction factor was predicted. Curvilinear membrane geometry induces transient anomalous diffusion. Finite values exist for the reduction of the diffusion coefficient in both the short- and the long-time limits. For symmetrically permeable cristae junctions, these values can be well approximated from projected membrane surface areas. If neglected, geometrical effects could lead to incorrect interpretation of experimental results. Hence, empirical measurements of diffusion in highly curved biological membranes should include geometrical information as a critical component of data analysis.

Mitochondria in most protists have tubular inner membranes. The evolutionary advantage of lamellar crista development could be the reduction of protein mobility within a mitochondrion, the formation of more stable complexes and thus more proteins per unit mitochondrial length. Further experimental research is needed to validate the consequences derived from these theoretical considerations.
